# Survey of Hand Hygiene, High-Touch Device Use, and Proper Habits of Health Care Workers for Infection Risk Prevention: Protocol for a Cross-Sectional Study

**DOI:** 10.2196/60450

**Published:** 2025-04-29

**Authors:** Antonio Aprile, Giustino Morlino, Claudia Mosconi, Luca Guarente, Alessandra Messina, Mariachiara Carestia, Patrizia De Filippis, Francesca Pica, Massimo Maurici, Leonardo Palombi

**Affiliations:** 1 Department of Experimental Medicine ​Faculty of Medicine and Surgery University of Rome (Tor Vergata) Rome Italy

**Keywords:** health care–associated infection, nosocomial infection, fomite, hand hygiene, universal precautions, infection prevention, infectious disease, handwashing, decontamination

## Abstract

**Background:**

Care-related infections are infectious diseases that occur in a care setting. The most important prevention methods are hand hygiene and the proper use of gloves and gowns. Recent literature points out that hand contact with mobile devices or potentially contaminated environments can promote an increased occurrence of health care–associated infections (HAIs).

**Objective:**

This study aims to analyze the correlation between the microbial population present on the hands of health care professionals in the wards of the Tor Vergata Hospital in Rome and the microorganisms present on the surfaces of their smartphones and tablets by searching for the main agents responsible for HAIs.

**Methods:**

Sterile swabs will be used to collect samples from hands and smartphones, which will then be plated on nutrient agar and other selective media for microbial count. Colonies showing growth and morphological characteristics suggestive of potential pathogens will be isolated and subjected to further analysis for identification at the species level and for antimicrobial resistance profiling (using a proprietary automated analytical profile index system). Sampling will be conducted quarterly (first 2 weeks of each quarter) to assess any changes in microbial flora. In parallel, an Italian version of the World Health Organization questionnaire on health care workers’ knowledge of hand hygiene and a questionnaire on the use of high-touch devices will be administered to participants. Each quarter, 30 health workers will be selected, resulting in a total of 120 health workers and 240 samples collected by the end of the study. For each sample, the analysis will focus on quantifying the total bacterial load at 37 °C and 22 °C, along with detecting coliforms or *Escherichia coli*, *Enterococci*, *Staphylococci*, *Acinetobacter*, *Klebsiella*, *Pseudomonas*, and any associated antimicrobial resistance.

**Results:**

The study aims to begin sample collection by June 2025. The protocol was properly evaluated and approved by the territorial ethics committee “Lazio Area 2” on March 21, 2024, with the code 76.24 CET2 utv_ptv.

**Conclusions:**

The findings of this study will be crucial in highlighting the need for targeted education and training of health care practitioners involved in the study, with a focus on the prevention of HAIs.

**International Registered Report Identifier (IRRID):**

PRR1-10.2196/60450

## Introduction

Health care–associated infections (HAIs) are infectious diseases that occur in a health care setting, both in hospitals and all health care facilities [1]. They are among the major complications related to care processes, causing prolonged hospital stays, aggravation of underlying pathologies, and long-term disabilities with consequent clinical, social, and economic impacts [2]. Moreover, it is estimated that HAIs are responsible for 16 million additional inpatient days and 37,000 deaths with HAI as a primary cause each year [3]. The Italian law 81/08 (which primarily aims to ensure and guarantee an adequate level of safety in the workplace) emphasizes the importance of responsibly managing infectious risk, including in the proper provision and use of protective clothing (including hospital gowns) as well as remarking on the importance of avoiding exposure of uniforms to the external environment [4]. Studies have shown suboptimal adherence to infection prevention protocols among health care workers, with hand hygiene compliance ranging from 7.7% to 89.7% [5].

**Figure 1:**
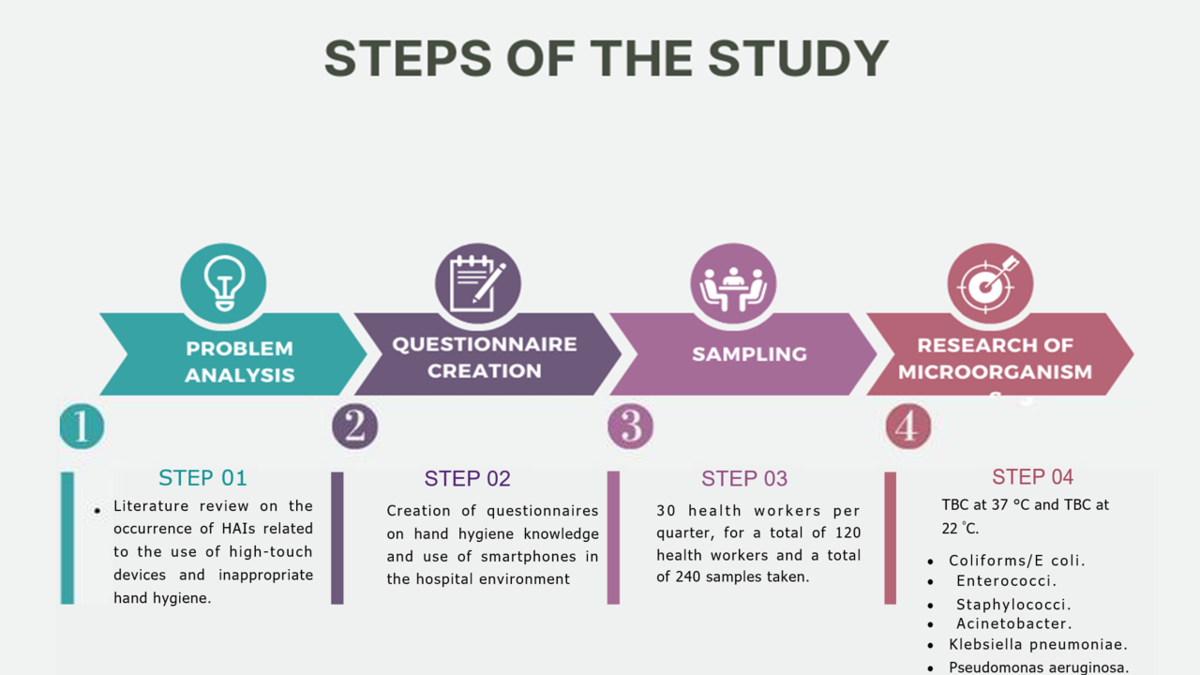
Steps of the study. HAIs: health care–associated infections; TBC: total bacterial count.

The “Regional Hand Hygiene Intervention Plan” from the Lazio Region highlights handwashing as the most effective intervention for the prevention of HAIs, although it notes suboptimal adherence in care facilities to this correct practice [6]. Another important prevention method concerns the correct use of gloves, which in no way replaces the need to wash hands; moreover, their incorrect use can increase the risk of pathogenic germ transmission from caregiver to patient or vice versa [7]. In addition, over the past 10 years, there has been widespread use of smartphones in the workplace among health care workers [8-10]. These high-touch devices have become integral to health care practice, serving as valuable tools for disease diagnosis, drug reference, medical calculators, and communication among health care teams [11, Figure 1]. However, the constant contact with hands and potentially contaminated environments, coupled with the presence of a touch screen, makes smartphones potential vectors for microbial transmission [12-15]. Studies have revealed the presence of various pathogens, including multidrug-resistant bacteria, on the surfaces of smartphones used by health care workers [16-18]. Notably, contamination is not limited to the touch screen; the posterior surface of smartphones has also been found to harbor significant bacterial contamination [19]. These findings underscore the potential role of smartphones in the spread of nosocomial infections, particularly in settings where appropriate preventive measures, such as proper hand hygiene and disinfection of the cell phone surface, are lacking [20-26]. As evidence of this, several studies have shown that 58% of staff reported cleaning their phones once a day, at best [27], and that a range of 44%-90% do not clean their devices at all [28]. There are also early studies referring to Italy that would support the need to investigate this dynamic further, although they were limited to analyzing the phenomenon in individual departments [29-31]. The purpose of our study is, therefore, to analyze the correlation between the microbial population present on the handprints of health care professionals in the wards of the Tor Vergata Hospital in Rome, which will serve as a pilot for this study, and the microorganisms present on the surfaces of workers’ smartphones and tablets by searching for the main agents responsible for HAIs, promoting the use of modern surveillance platforms for nosocomial infections. This will allow for a more accurate evaluation of all possible prophylaxis interventions.

This study, conducted by the Department of Biomedicine and Prevention, Section of Hygiene and Preventive Medicine, University of Rome Tor Vergata, is part of the Digital Lifelong Prevention (DARE) project (code PNC_0000002 - CUP: B33C22001340002) and specifically will contribute to the collection of data needed to carry out the activities of pilot 1: “Data mining and AI approaches to predict/prevent risk of Healthcare Associated Infections (HAIs),” under work package 3, task 3.1 (Digital Tools in Children and Aged Frail Subjects).

## Methods

### Recruitment

Participants in the study will be selected voluntarily from a population of health care workers (nurses, doctors, and health care assistants) who use high-touch devices in the very high (eg, intensive care unit), high (eg, surgical wards), and medium (eg, general medicine) infectious risk departments. The division of the wards into very high risk, high risk, and medium risk will be based on the detailed analyses carried out at Tor Vergata Polyclinic in a recent study [32]. Participants who do not use a smartphone or tablet or who do not have them at the time of the study will be excluded from the sampling. Recruitment of participants will be voluntary from among health workers in the departments involved. Both sample collection and questionnaire administration will be carried out in a selected room of the health worker’s department. The surveys will be conducted during the afternoon shift change (at 2 PM). This study will last 1 year, and sampling will be performed throughout. In the participating hospital, 30 health care workers per quarter will be selected, for a total of 120 health care workers and a total of 240 samples taken; alert microorganisms will be tested for on each sample.

### Staff

Staff involved in the project will include nurses and prevention technicians who will be responsible for taking samples. Biologists and laboratory medicine residents will also be involved in the laboratory analyses in support of the teaching staff of the Department of Biomedicine and Prevention, Section of Hygiene and Preventive Medicine of Rome Tor Vergata. The physicians specializing in hygiene and preventive medicine will be in charge of collecting questionnaires and analyzing the collected data, supported by the teaching staff.

### Privacy Policy

The following privacy policy will be given to each recruited health professional:

*Dear Participant,*

We wish to inform you that the GDPR (EU Regulation No. 2016/679) provides for the protection of individuals with regard to the processing of personal data.

According to the indicated regulation, such processing will be based on the principles of lawfulness, fairness and transparency, adequacy, relevance and limitation, accuracy and updating, non-excessiveness, and accountability.

Information on the processing of your data:

We are conducting a research project called “Survey on hand hygiene, use of high touch devices and correct habits by health care workers for the prevention of infectious risk.” The aim of this research is to better understand the handwashing and device usage habits of healthcare workers. To do this, we will be taking swabs of your hands and your device, and you will also be asked to fill out a questionnaire.

Your participation in this research is completely voluntary, and there are no consequences if you choose not to provide your data. However, providing your data is essential for us to conduct this study, so if you decline, you won't be able to participate.

We take data protection very seriously and will implement all necessary measures to safeguard your information and rights. Your data will not be shared with any third parties, and any results published will be anonymized and aggregated.

You have the right to decide whether or not to give your consent for us to use your data after reading this information.

### Questionnaire

During the collection of samples, the validated World Health Organization questionnaire, translated into Italian and adapted for the Lazio Region, on hand hygiene will be administered to all participants to collect information on sociodemographic characteristics (age, gender, profession, and department). A short questionnaire will be administered with questions on the use of high-touch devices and personal protective equipment (PPE; eg, gloves, gowns) in the workplace. Personal and sensitive data of the participants in the study will not be collected. Questionnaires can be found in Multimedia Appendix 1. Both questionnaires will be completed in the presence of the researchers and consist of closed-ended questions on a 7-point Likert scale.

### Sample Collection and Laboratory Testing

Samples, both from hands and smartphone touch screens, will be taken using sterile nylon-tipped flocked swabs (Copan SRK Elution Flocked Swabs, Biogenetics Diagnostics srl, Ponte San Nicolò PD, Italy), applying methods for environmental microbiology for small surfaces [33,34]. The swabs will be supplied with 2.5 mL of a sterile surface recovery kit transport solution (Letheen Broth Solution, a nutrient and neutralizing solution), in which the microorganisms taken/flocked with the swab are resuspended. This solution is useful for preserving the bacteria and neutralizing the activity of possible disinfectants on the surfaces.

Samples will be taken by properly trained, qualified staff capable of complying with all asepsis regulations. Hand sampling will be performed for each participant on all fingertips and palms, always with the same swab for both hands. A swab will be used for each smartphone that will be swiped on both the anterior (touch screen) and posterior parts.

After the sampling session and immediately after delivery of the completed questionnaire, the samples will be transferred to the Laboratory of Environmental Microbiology, Department of Biomedicine and Prevention, Tor Vergata University, for subsequent analysis, which will be performed according to current microbiological procedures.

Each questionnaire is assigned a sequential number, which is written on the corresponding swab tube.

The analysis includes a quantitative and qualitative assessment, searching the heterotrophic plate total bacterial count (HTBC) at 37 °C and 22 °C for coliforms or *Escherichia coli* (*E coli*), *Enterococci*, *Staphylococci*, Gram-negative bacteria, *Pseudomonas aeruginosa*, and *Mycetes*. In addition, the isolated strains will be typed through standardized miniaturized tunnels for biochemical analytical profile index tests. We decided to select the microorganisms that are most frequently associated with the occurrence of HAIs [35,36]. Sampling is carried out immediately after delivery of the completed questionnaire, using a swab and a tube containing 2.5 mL of transport solution. Each questionnaire is assigned a sequential number, which is written on the corresponding swab tube. The standard sampling procedure for environmental surface sampling will be used.

The swabs are transferred to the laboratory immediately after sampling and are then seeded as follows. For the determination of both HTBC at 37 °C and 22 °C, the bulk seeding technique is used with 500 µl per sample on plates of a tryptone soy agar medium, which are then placed in a thermostat at 37 °C and 22 °C for 48 hours and 72 hours, respectively. Per sample, 250 µl are distributed and spatulated on a plate with a Harlequin *E coli*/coliform chromogenic medium for the determination of *E coli* and total coliforms; 250 µl are distributed and spatulated on a sterile Slanetz and Bartley agar plate for the determination of *Enterococci*; 250 µl are distributed and spatulated on a plate containing a Baird Parker agar medium for the determination of *Staphylococci*; 250 µl are distributed and spatulated on a plate with MacConkey Agar medium for the determination of Gram-negative bacteria; 250 µl are distributed and spatulated on a plate with a cetrimide agar base medium for the determination of *Pseudomonas*; and 250 µl are distributed and spatulated on a plate with a Sabouraud medium for the determination of *Mycetes* (yeasts and microfungi). All plates are then transferred to a thermostat at 37 °C for 24-48 hours.

After the appropriate culture times, the plates are observed, and a count of the colonies grown is made. The results obtained, expressed as colony-forming units (CFUs)/dm^2^, will be recorded on a table (Multimedia Appendix 2) that includes sample ID, date of sampling, and any observations. In addition to a quantitative evaluation, a qualitative evaluation will be carried out based on the observation of colony morphology, and after the selection of the different types, the identification of individual types will be preceded by a specific miniaturized biochemical test (analytical profile index test) following the procedure indicated by the manufacturer. Colonies in each treated plate will be screened for resistance by using broad-spectrum antibiotics: 10 μg ampicillin, 4 μg gentamicin, and 5 μg chloramphenicol, placed separately on Muller-Hinton agar plates and incubated in aerobiosis at 37 °C for 18 hours.

Minimum inhibitory concentration will be performed using the automatic ID/AST Vitek 2 Compact instrument (Biomerieux, France) and Gram-positive and Gram-negative susceptibility test cards (AST-P577 and AST-N117; Biomerieux, France).

The diffusion method according to Kirby-Bauer [15,18,27] will be used for the antibiotics aztreonam, minocycline, and levofloxacin. Breakpoints will be interpreted according to Clinical and Laboratory Standards Institute guidelines [37]. This type of investigation will have a quarterly periodicity that may allow us to assess any quantitative and qualitative changes in the microbial flora at different times of the year due to seasonal differences in temperature and humidity.

### Data Recording

The questionnaire responses, quantitative data from plate reading, annotations made on the sampling form, and results of bacterial identifications will be recorded on an Excel Professional Plus 2016 (Italian language; Microsoft Corporation) worksheet.

### Statistical Analysis

A descriptive statistical analysis will be conducted to outline the sociodemographic data of the healthcare worker sample and the responses to the questionnaires, aimed at determining the frequency of high-touch device and PPE use and awareness of contamination. Subsequently, inferential investigations will be carried out between the sociodemographic data of the sample and the results from the microbiological swabs. The results for the descriptive section of the questionnaire will be expressed in terms of numbers and percentages. As for the quantitative microbiological results, these will be expressed as means (SDs) and medians (IQRs), as it is expected that the distribution of some variables will not be Gaussian-like. The microbiological results will be presented by stratifying against the questionnaire variables. For the qualitative analysis, we will construct a heat map with R software (version 4.5.0; R Foundation for Statistical Computing) to determine which continuous variables will be transformed into dichotomous variables, using a threshold of 100 CFUs/dm^2^ as indicated in recent literature [12], so that dichotomous and continuous variables can be compared on a single map. For the species under investigation, we will describe the frequency of occurrence. To graphically show the relationship between the questionnaire variables of interest and bacterial concentrations, scatter plots will be constructed expressing the bacterial concentrations on a logarithmic scale (lnCFU/dm^2^), color differentiating and constructing different CIs in the graph according to the categorical variable (eg, different cleaning methods, frequency of training, gender). Box-and-whisker graphs will also be constructed to show the distributions of the HTBC at 37 °C and 22 °C, and for specific agents according to the above categorical variables. Quantitative analyses will be performed using SPSS version 22.0 (IBM Corp), verifying the normality of continuous variables using the Shapiro-Wilk test and performing parametric (*t* test) or nonparametric (Kruskal-Wallis) tests depending on the results of this analysis. Samples will be compared according to the following variables: gender, type of ward, cleaning frequency, cleaning method, type of telephone case, and gown use. Subsequently, variables with a significance of *P*<.20 will be selected for multiple linear regression models to describe the relationships between quantitative and categorical microbiological variables.

### Steps of the Study

The following steps will be followed for this study:

1. A total of 30 health professionals from the University Polyclinic of Rome Tor Vergata will be selected voluntarily and will undergo both hand and high-touch device sampling for a total of 2 samples per professional.
2. The two questionnaires will be submitted at the same time.
3. Laboratory and statistical investigations of the samples and responses to the questionnaires will be carried out.
4. This procedure will be repeated on a seasonal basis for 1 year.

### Ethics Approval

The protocol was properly evaluated and approved by the territorial ethics committee “Lazio Area 2” on March 21, 2024, with the code 76.24 CET2 utv_ptv. Participation in this survey is entirely voluntary. The administration of the questionnaire and the collection of any samples will only commence after participants have provided their informed consent. All data collected will be processed in strict accordance with current privacy regulations (further details can be found in the dedicated ‘Privacy Policy’ section). The information obtained from this study will be published in an anonymized and aggregated form, ensuring that individual responses cannot be identified, and it will not be shared with any third parties. It is important to note that there are no forms of compensation offered to participants or those submitting the questionnaire for their involvement.

## Results

The study aims to start sample and questionnaire data collection in June 2025 and continue with the collection until the following year.

The results may be published in national and international journals, which will be led by the University of Rome Tor Vergata once the trial is over. The results, even if negative, will be shared with the scientific community.

## Discussion

Our study aims to identify the main pathogens responsible for HAIs and to promote the use of modern surveillance platforms to monitor and prevent these infections.

The research is set in a context where HAIs represent a major public health challenge with significant clinical, social, and economic impact. Although the importance of hand hygiene and the proper use of PPE in the prevention of HAIs is widely recognized, adherence to these practices is often suboptimal in health care facilities.

This study aims to investigate the correlation between the microbial flora present on health care workers’ hands and that present on their electronic devices. The expected results of the study could contribute to a better understanding of the role of electronic devices in the transmission of HAIs and promote the adoption of more effective preventive measures. The expected results of the study could contribute to a better understanding of the role of electronic devices in the transmission of HAIs and promote the adoption of more effective preventive measures.

It would be interesting to assess the impact of the health care environment on the contamination of electronic devices. To this end, it could be hypothesized that wards with a high prevalence of multidrug-resistant pathogens have higher device contamination than those with a lower prevalence.

It is also worth evaluating the effectiveness of various preventive measures, such as hand hygiene, disinfection of device surfaces, and the use of protective cases, in reducing the contamination of electronic devices.

Another important consideration is to analyze the influence of health care workers’ behavior on the contamination of electronic devices. For example, it could be hypothesized that health care workers who use the devices during treatment procedures have a higher contamination rate than those who only use them during breaks. However, the study has some limitations. First, the sample size (120 health care workers) may not be large enough to guarantee the generalizability of the results. Second, the study focuses on a single hospital, which may limit the applicability of the results to other health care settings. In conclusion, this study represents an important contribution to research on HAIs and the role of electronic devices in their transmission. The expected results could provide useful information for the development of more effective prevention strategies and the promotion of a more conscious use of electronic devices by health care professionals.

The purpose of the study is to emphasize the importance of proper hand hygiene and proper use of high-touch devices and PPE during work for the prevention of HAIs. If the results highlight the presence of multiple alert organisms, it will be important in a follow-up study to promote educating staff on the prevention of HAIs. Following this, new methods of monitoring could be implemented to improve adherence to good practices for preventing HAIs.

## Appendix

Multimedia Appendix 1 Hand hygiene questionnaire.

Multimedia Appendix 2 Positivity of microorganisms in analyzed samples table.

## Abbreviations

CFU: colony-forming unit
DARE: Digital Lifelong Prevention
HAI: health care–associated infection
HTBC: heterotrophic plate total bacterial count
PPE: personal protective equipment

## Data Availability

The datasets generated during or analyzed during this study will be available from the corresponding author upon reasonable request.
