# Supplementary material for: Survey of Hand Hygiene, High-Touch Device Use, and Proper Habits of Health Care Workers for Infection Risk Prevention: Protocol for a Cross-Sectional Study
Source: JMIR Res Protoc. 2025 Apr 29;14:e60450. doi: 10.2196/60450 (PMC12076024; doi:10.2196/60450)
Supplement: Multimedia Appendix 1 [file resprot_v14i1e60450_app1.pdf]

PIANO DI INTERVENTO REGIONALE SULL'IGIENE DELLE MANI

Questionario sulla percezione degli operatori sanitari

Visto che la tua attività professionale di pone a contatto diretto e quotidiano con i pazienti, siamo interessati a conoscere la tua **opinione** sulle infezioni correlate all'assistenza e sull'igiene delle mani.

La compilazione del questionario richiede circa 10 minuti. Ti chiediamo di:

- Scegliere una sola risposta per ogni domanda
- Per le risposte che riportano una scala di valori (ad es. da NON EFFICACE a MOLTO EFFICACE) ti chiediamo di mettere una croce sulla casella che rappresenta meglio la tua opinione fra i due estremi
- Leggere attentamente le domande prima di rispondere
- Le risposte e i risultati dei questionari saranno riservate e analizzate in forma aggregata

Breve glossario

**Frizione**	Applicazione di una prodotto a base alcolica per ridurre o inibire la proliferazione dei microrganismi senza ricorrere a fonti idriche esterne e senza risciacquo né asciugatura con asciugamani o altro.
**ICA**	Infezioni Correlate all'Assistenza
**Lavaggio delle mani**	Lavaggio delle mani con acqua e sapone o altri detergenti con agenti antisettici.
**Prodotto a base alcolica**	Una preparazione contenente alcol (liquida, gel o schiuma) ideata per essere applicata sulle mani allo scopo di ridurre la crescita dei microorganismi. Queste preparazioni possono contenere uno o più tipi di alcol assieme a eccipienti, altri ingredienti attivi e umettanti.

<u>**NB**</u>: Il materiale presentato è stato tradotto da documenti OMS e adattato al contesto regionale. L'OMS non è responsabile dei contenuti e dell'accuratezza delle traduzioni e degli adattamenti effettuati. Per la consultazione dei documenti originali si rimanda all'indirizzo http://www.who.int/gpsc/5may/tools/en/index.html

1	**DATA DI COMPILAZIONE** (GG/MM/AAAA)	___ / ___ / ________
2	**STRUTTURA**	
3	**REPARTO/AMBULATORIO**	
4	**SESSO**	☐ F<br>☐ M
5	**ETÀ** (NN)	_____
6	**ANZIANITÀ LAVORATIVA** (NN)	_____
7	**QUALIFICA**	☐ Infermiera/e<br>☐ Ostetrica/o<br>☐ Medico<br>☐ Studente medicina<br>☐ Terapista<br>☐ Altro (specificare) ____________________________<br>☐ Operatore di supporto<br>☐ Studente scienze infermieristiche/ostetriche<br>☐ Specialista in formazione<br>☐ Tecnico
8	**AREA ASSISTENZIALE**	☐ Medicina interna<br>☐ Chirurgia<br>☐ Terapia intensiva<br>☐ Emergenza<br>☐ Ostetricia/Ginecologia<br>☐ Pediatria/Neonatologia<br>☐ Riabilitazione/Lungodegenza<br>☐ Attività ambulatoriale<br>☐ Altro (specificare) ____________________________
9	**NEGLI ULTIMI TRE ANNI SEI STATO FORMATO SULL'IGIENE DELLE MANI?**	☐ SÌ<br>☐ NO
10	**USI REGOLARMENTE SOLUZIONI A BASE ALCOLICA PER L'IGIENE DELLE MANI?**	☐ SÌ<br>☐ NO
11	**QUAL È, SECONDO TE, LA PERCENTUALE MEDIA DI PAZIENTI CHE SVILUPPANO UNA ICA?**	_____ %<br>☐ Non so
12	**IN GENERALE, QUALE RITIENI CHE SIA L'IMPATTO DELLE ICA SUGLI ESITI CLINICI DEL PAZIENTE?**	☐ molto basso<br>☐ basso<br>☐ alto<br>☐ molto alto
13 *16*	**QUAL È L'EFFICACIA DELL'IGIENE DELLE MANI NELLA PREVENZIONE DELLE ICA?**	☐ molto basso<br>☐ basso<br>☐ alto<br>☐ molto alto
14	**QUAL È L'IMPORTANZA CHE LA TUA STRUTTURA DÀ ALL'IGIENE DELLE MANI?**	☐ bassa priorità<br>☐ media priorità<br>☐ alta priorità<br>☐ priorità molto alta
15	**SECONDO TE QUAL È LA PERCENTUALE MEDIA DI SITUAZIONI IN CUI GLI OPERATORI SANITARI DELLA TUA STRUTTURA PRATICANO L'IGIENE DELLE MANI QUANDO È RICHIESTA?**	_____ %<br>☐ Non so

16	**SECONDO TE QUALI, FRA LE AZIONI DI SEGUITO ELENCATE, SONO PIÙ EFFICACI PER MIGLIORARE IN MODO PERMAMENTE LA PRATICA DELLA IGIENE DELLE MANI NELLA TUA STRUTTURA?**	
	a) Supporto della Direzione e dei Responsabili UUOO e Coordinatori infermieristici nella promozione dell'igiene delle mani:<br>NON EFFICACE ☐ ☐ ☐ ☐ ☐ ☐ ☐ MOLTO EFFICACE<br>b) Disponibilità di soluzione alcolica a ogni punto di assistenza:<br>NON EFFICACE ☐ ☐ ☐ ☐ ☐ ☐ ☐ MOLTO EFFICACE<br>c) Manifesti e altri promemoria affissi nei punti di assistenza:<br>NON EFFICACE ☐ ☐ ☐ ☐ ☐ ☐ ☐ MOLTO EFFICACE<br>d) Formazione specifica per ogni operatore sanitario:<br>NON EFFICACE ☐ ☐ ☐ ☐ ☐ ☐ ☐ MOLTO EFFICACE<br>e) Istruzioni chiare e semplici sull'igiene delle mani sono visibili a ogni operatore sanitario:<br>NON EFFICACE ☐ ☐ ☐ ☐ ☐ ☐ ☐ MOLTO EFFICACE<br>f) Fornire un feedback regolare agli operatori sui loro comportamenti:<br>NON EFFICACE ☐ ☐ ☐ ☐ ☐ ☐ ☐ MOLTO EFFICACE<br>g) Praticare sempre l'igiene delle mani e fare da buon esempio ai colleghi:<br>NON EFFICACE ☐ ☐ ☐ ☐ ☐ ☐ ☐ MOLTO EFFICACE<br>h) Invitare i pazienti a ricordare agli operatori di praticare l'igiene delle mani:<br>NON EFFICACE ☐ ☐ ☐ ☐ ☐ ☐ ☐ MOLTO EFFICACE	
17	**QUALE IMPORTANZA VIENE DATA DAL RESPONSABILE DEL TUO REPARTO AL FATTO CHE TU PRATICHI L'IGIENE DELLE MANI?**	
	NESSUNA IMPORTANZA ☐ ☐ ☐ ☐ ☐ ☐ ☐ MOLTISSIMA IMPORTANZA	
18	**QUALE IMPORTANZA VIENE DATA DAI TUOI COLLEGHI AL FATTO CHE TU PRATICHI L'IGIENE DELLE MANI?**	
	NESSUNA IMPORTANZA ☐ ☐ ☐ ☐ ☐ ☐ ☐ MOLTISSIMA IMPORTANZA	
19	**QUALE IMPORTANZA VIENE DATA DAI PAZIENTI AL FATTO CHE TU PRATICHI L'IGIENE DELLE MANI?**	
	NESSUNA IMPORTANZA ☐ ☐ ☐ ☐ ☐ ☐ ☐ MOLTISSIMA IMPORTANZA	
20	**QUALE SFORZO DEVI FARE PER PRATICARE CORRETTAMENTE L'IGIENE DELLE MANI?**	
	NESSUNO SFORZO ☐ ☐ ☐ ☐ ☐ ☐ ☐ UN GRANDE SFORZO	
21	**SECONDO TE QUAL È, IN MEDIA, LA PERCENTUALE DI SITUAZIONI IN CUI PRATICHI CORRETTAMENTE L'IGIENE DELLE MANI?**	____ %

Grazie per il suo tempo!
