# Supplementary material for: Survey of Hand Hygiene, High-Touch Device Use, and Proper Habits of Health Care Workers for Infection Risk Prevention: Protocol for a Cross-Sectional Study
Source: JMIR Res Protoc. 2025 Apr 29;14:e60450. doi: 10.2196/60450 (PMC12076024; doi:10.2196/60450)
Supplement: Multimedia Appendix 2 [file resprot_v14i1e60450_app2.docx]

Table 1. Positivity of microorganisms in analyzed samples.

| sample | date | HBC 37°C | HBC  22°C | Acineto | E.Coli | Entero | Klebsiella | Stafilo | Pseudomonas |
| --- | --- | --- | --- | --- | --- | --- | --- | --- | --- |
| ID 1 | | | | | | | | | |
| Hands |  |  |  |  |  |  |  |  |  |
| Devices |  |  |  |  |  |  |  |  |  |
| ID 2 | | | | | | | | | |
| Hands |  |  |  |  |  |  |  |  |  |
| Devices |  |  |  |  |  |  |  |  |  |
| ID 3 | | | | | | | | | |
| Hands |  |  |  |  |  |  |  |  |  |
| Devices |  |  |  |  |  |  |  |  |  |
| ID 4 | | | | | | | | | |
| Hands |  |  |  |  |  |  |  |  |  |
| Devices |  |  |  |  |  |  |  |  |  |
| ID 5 | | | | | | | | | |
| Hands |  |  |  |  |  |  |  |  |  |
| Devices |  |  |  |  |  |  |  |  |  |
